# Decoding the metabolic cipher of dormant cancer cells: molecular mechanisms and therapeutic potentials

**DOI:** 10.1186/s12964-026-02933-w

**Published:** 2026-05-07

**Authors:** Yuao Qin, Junling Zhang, Yuyang Xiao, Yikai Zhang, Wenbin Liu, Xiangjian Luo

**Affiliations:** 1 Hunan Key Laboratory of Oncotarget Gene, Hunan Cancer Hospital and the Affiliated Cancer Hospital of Xiangya School of Medicine, Central South University, Changsha, Hunan 410013 PR China; 2 Key Laboratory of Carcinogenesis and Invasion, Chinese Ministry of Education, Cancer Research Institute, Xiangya School of Basic Medical Science, Central South University, Changsha, Hunan 410078 PR China; 3 Department of Otolaryngology, Head and Neck Surgery, Xiangya Hospital, Central South University, Changsha, Hunan 410008 PR China; 4 Department of Pathology, Hunan Cancer Hospital and The Affiliated Cancer Hospital of Xiangya School of Medicine, Central South University, Changsha, Hunan 410013 PR China; 5 Key Laboratory of Biological Nanotechnology of National Health Commission, Central South University, Changsha, Hunan 410078 China

**Keywords:** Dormant cancer cells, Metabolic reprogramming, Cancer recurrence, Drug resistance, Targeted therapy

## Abstract

Dormant cancer cells (DCCs) are non-proliferative cancer cells that enter cell cycle arrest in the G0–G1 phase and are recognized as a major cause of therapeutic resistance and cancer recurrence. In response to various intracellular and extracellular signals, DCCs undergo cellular reprogramming that confers drug resistance and enables them to evade immune surveillance. Once reactivated, these cells can resume proliferation, ultimately leading to tumor relapse. Metabolic reprogramming allows DCCs to adapt to the nutrient-deprived tumor microenvironment (TME), reduce energy consumption, and maintain redox homeostasis. Targeting these metabolic vulnerabilities provides promising opportunities to control recurrence and improve therapeutic outcomes. However, the metabolic reprogramming of DCCs is highly heterogeneous, which poses a major challenge for their complete eradication. In this review, we summarize the metabolic features of DCCs, describe the molecular mechanisms underlying metabolic reprogramming across distinct DCC subtypes, elucidate the interactive networks among distinct metabolic pathways, and discuss therapeutic strategies targeting metabolism of DCCs, with the goal of providing new insights into improving treatment efficacy and preventing recurrence.

## Introduction

Dormancy refers to a period in which the biological activities of an organism are temporarily suspended, functioning as an evolutionarily conserved strategy that promotes adaptation to stress and survival under adverse conditions [1]. The concept of “dormancy” was first introduced into oncology by Rupert Willis in 1934, who suggested that in patients without local recurrence, the presence of late metastases indicated that “tumor cells must remain in a dormant state within the tissues where they are suppressed” [2]. In 1954, Geoffrey Hadfield further proposed that late metastatic relapse is driven by dormant cancer cells (DCCs) undergoing “temporary mitotic arrest” [3]. Today, it is widely recognized that DCCs are non-proliferative cancer cells that have entered G0–G1 cell cycle arrest [4].

With increasing research, DCCs have been shown to follow a distinctive life cycle: they first locate and occupy a niche, interact with various microenvironmental factors, then undergo G0–G1 cell cycle arrest and cellular reprogramming to adapt to the niche and evade immune surveillance, thereby establishing long-term dormancy [5]. Importantly, DCCs can also be reactivated by intracellular or extracellular signals, leading to metastatic recurrence [5]. The entry of cancer cells into dormancy is regulated by multiple key proteins, including dual-specificity tyrosine phosphorylation-regulated kinase 1 A (DYRK1A) [6], the cyclin-dependent kinase inhibitors p21 and p27 [7], the balance between p38 mitogen-activated protein kinase (p38 MAPK) and extracellular signal-regulated kinases 1/2 (ERK1/2) [8], growth arrest-specific protein 6 (GAS6) [9], and bone morphogenetic proteins 4 and 7 (BMP4/7) [10, 11]. Among these, p38 MAPK ^high^ /ERK ^low^, along with elevated p21 and p27 levels, are widely regarded as canonical markers of DCCs.

To avoid ambiguity in terminology, several overlapping concepts require explicit clarification herein. Broadly, quiescent cells refer to any cell population in a reversible, non-proliferative state in the G0 phase of the cell cycle, encompassing both normal somatic cells (quiescent stem cells) and malignant cells (DCCs) [12, 13]. Slow-cycling cells share a core definitional framework with quiescent cells, and in the context of malignant disease, the terms “slow-cycling”, “dormant”, and “quiescent” are often used interchangeably in the existing literature [14]. Drug-tolerant persister (DTP) cells are defined as a subpopulation of cancer cells that enter a reversible quiescent or slow-cycling state, with blunted responsiveness to external stimuli. These cells tolerate exposure to anticancer drugs via the interplay of multiple underlying mechanisms, and resume growth and proliferation upon drug withdrawal, ultimately driving treatment resistance and cancer recurrence [15]. To date, there are no universally accepted biomarkers for the definitive identification of DTP cells, and the field continues to debate whether the definitions of DTP cells and DCCs are synonymous [16–18]. One possible explanation is that DTP cells represent a heterogeneous collective encompassing drug-tolerant DCCs, cancer stem cells (CSCs), and other cancer cell subpopulations, while not all DCCs or CSCs exhibit the phenotypic hallmarks of DTP cells [19–21].

The study of cancer metabolism dates back to the pioneering work of Otto Warburg in the 1920s, who discovered that cancer cells preferentially “ferment” glucose into lactate even in the presence of oxygen, a phenomenon later termed the Warburg effect [22]. Subsequent research has revealed that cancer cell metabolism is highly plastic and heterogeneous, evolving dynamically throughout tumor progression, particularly in the transition between proliferative cancer cells and DCCs [23]. Through metabolic reprogramming, cancer cells adapt to the tumor microenvironment (TME), satisfy intrinsic energy demands, maintain redox balance, and acquire drug resistance, thereby sustaining long-term dormancy [24]. Likewise, the reactivation of DCCs also critically depends on metabolic reprogramming [24].

Although cancer metabolism has been extensively investigated [25], research on the metabolic mechanisms of DCCs is still in its infancy, with many unresolved questions. In this review, we systematically summarize the characteristics and molecular mechanisms of DCC metabolism in glucose, lipid, amino acid, and nucleotide pathways, elucidate the interplay and cross-regulation among these metabolic processes, and highlight the heterogeneity and complexity of metabolism in DCCs. Building on this foundation, we discuss key metabolic targets, explore potential therapeutic strategies against DCCs, evaluate the limitations of current approaches, and outline future perspectives for precision cancer therapy.

## Metabolic characteristics of dormant cancer cells

**Fig. 1:**
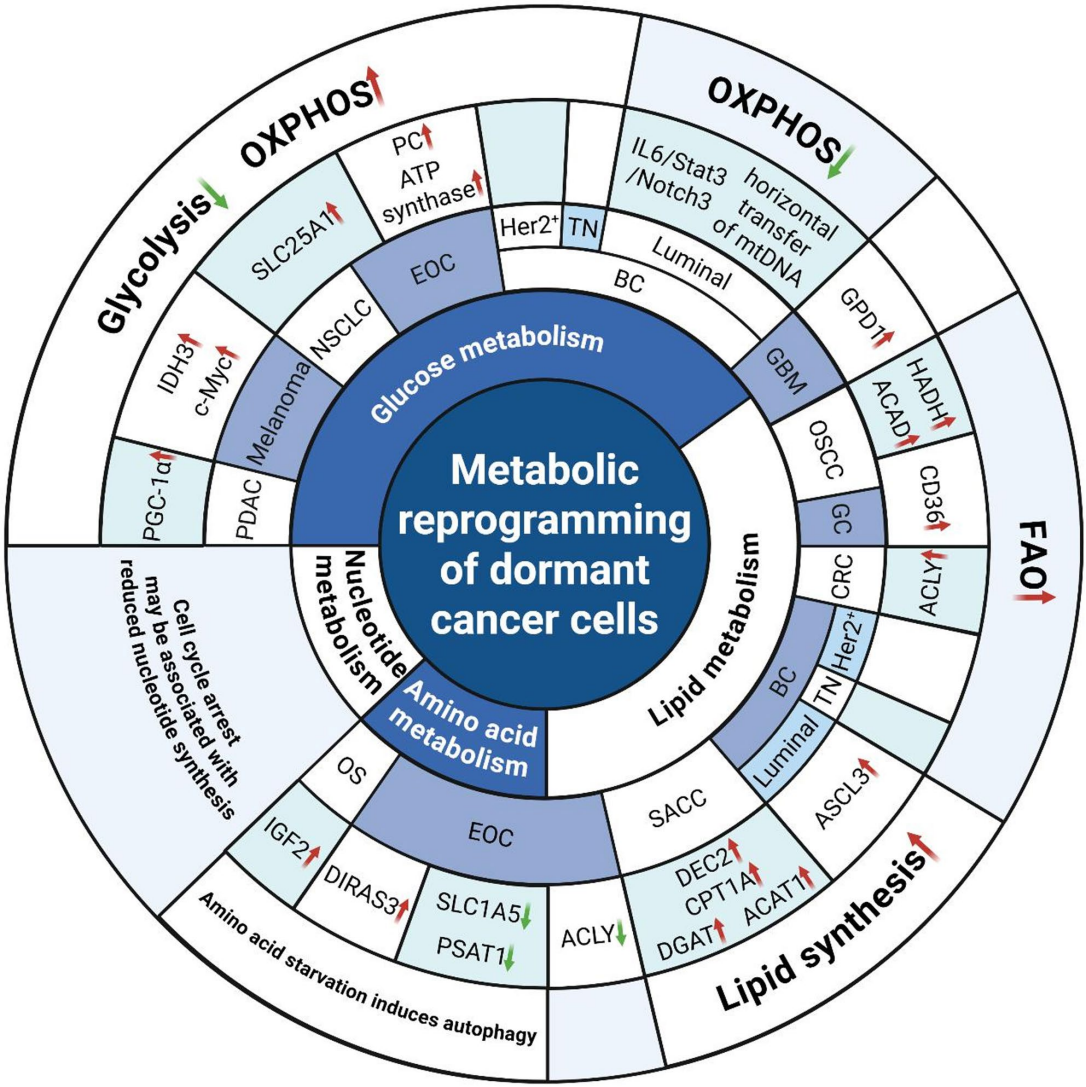
Metabolic reprogramming of dormant cancer cells. This figure illustrates the key molecules and signaling pathways implicated in the metabolic reprogramming of dormant cancer cells (DCCs) across four major pathways: glucose, lipid, amino acid, and nucleotide metabolism. It summarizes the distinct metabolic features of DCCs within each pathway and highlights the heterogeneity and complexity of their metabolic adaptations. (Red arrows indicate upregulation/enhancement, whereas green arrows indicate downregulation/reduction.) Abbreviations: PDAC, pancreatic ductal adenocarcinoma; NSCLC, non-small cell lung cancer; EOC, epithelial ovarian cancer; BC, breast cancer; TNBC, triple negative breast cancer; GBM, glioblastoma; OSCC, oral squamous cell carcinoma; GC, gastric cancer; CRC, colorectal cancer; SACC, salivary adenoid cystic carcinoma; OS, osteosarcoma

The metabolism of most DCCs is characterized by reduced glycolysis, elevated OXPHOS and FAO, autophagy triggered by amino acid deprivation, and diminished de novo nucleotide synthesis [26–29]. This metabolic profile allows DCCs to conserve energy, preserve redox balance, and persist in nutrient-limited environments [24, 30]. Nevertheless, a subset of DCCs exhibits alternative metabolic adaptations, such as suppressed OXPHOS or enhanced lipid biosynthesis [31, 32]. Such heterogeneity is shaped by the intrinsic metabolic state of cancer cells, TME, and dynamic metabolic reprogramming (Fig. 1). Once reactivated, cancer cells typically increase glycolysis and glutamine utilization, accompanied by markedly elevated uptake of nutrients—including glucose, fatty acids, and amino acids—to sustain their energy and biosynthetic requirements [24, 33]. These metabolic reprogramming enable cancer cells to circumvent nutrient limitations, thereby driving tumor growth and metastatic recurrence [30, 33]. In this section, we summarize the metabolic characteristics of DCCs in glucose, lipid, amino acid, and nucleotide metabolism, elucidate the molecular mechanisms underlying their metabolic reprogramming, and discuss the heterogeneity and complexity of metabolism in DCCs.

### Glucose metabolism

In the 1920s, Otto Warburg discovered that cancer cells predominantly utilize glycolysis for energy production under aerobic conditions, a phenomenon known as the Warburg effect [34]. As research has progressed, it has become increasingly evident that cancer cells also exploit mitochondrial oxidative phosphorylation (OXPHOS), which plays a critical role in tumor metabolism [35, 36]. It is now widely accepted that cancer cells exhibit a metabolic continuum, enabling them to concurrently utilize both glycolysis and OXPHOS. Through metabolic reprogramming, they dynamically regulate the balance between these two pathways to meet their energy demands across different cellular states and microenvironmental conditions [37, 38]. In most DCCs, the glucose metabolism is marked by reduced glycolysis and enhanced mitochondrial OXPHOS [26, 39, 40]. The oncogenic mutation Kras^G12D^ is a critical driver of glucose metabolism, redirecting glycolytic intermediates into the hexosamine biosynthetic pathway (HBP) and the pentose phosphate pathway (PPP) [41]. Viale et al. developed a tetracycline-inducible Kras^G12D^ transgenic mouse model of pancreatic ductal adenocarcinoma (PDAC) [26]. Following doxycycline withdrawal, pancreatic tumors regressed, yet a subset of DCCs persisted and could give rise to tumor recurrence upon reactivation [26]. Compared with proliferative PDAC cells, DCCs exhibit markedly elevated levels of peroxisome proliferator-activated receptor gamma coactivator-1 alpha (PGC-1α) [26], a master regulator of mitochondrial biogenesis and function, including OXPHOS and reactive oxygen species (ROS) detoxification [42]. Further studies demonstrated that DCCs depend on OXPHOS for energy production, with no compensatory increase in glycolysis upon OXPHOS inhibition[26]. Similarly, Madonna et al. generated a Her2⁺ breast cancer model using the same strategy and employed two fluorescent probes — 2-[N-(7-nitrobenz-2-oxa-1,3-diazol-4-yl)amino]-2-deoxyglucose (2-NBDG) to measure glucose uptake and tetramethyl rhodamine ethyl ester (TMRE) to assess mitochondrial membrane potential [43]. Their findings corroborated the same conclusion: The balance between glycolysis and OXPHOS changed significantly in DCCs, namely reduced glycolysis and enhanced OXPHOS.

**Fig. 2:**
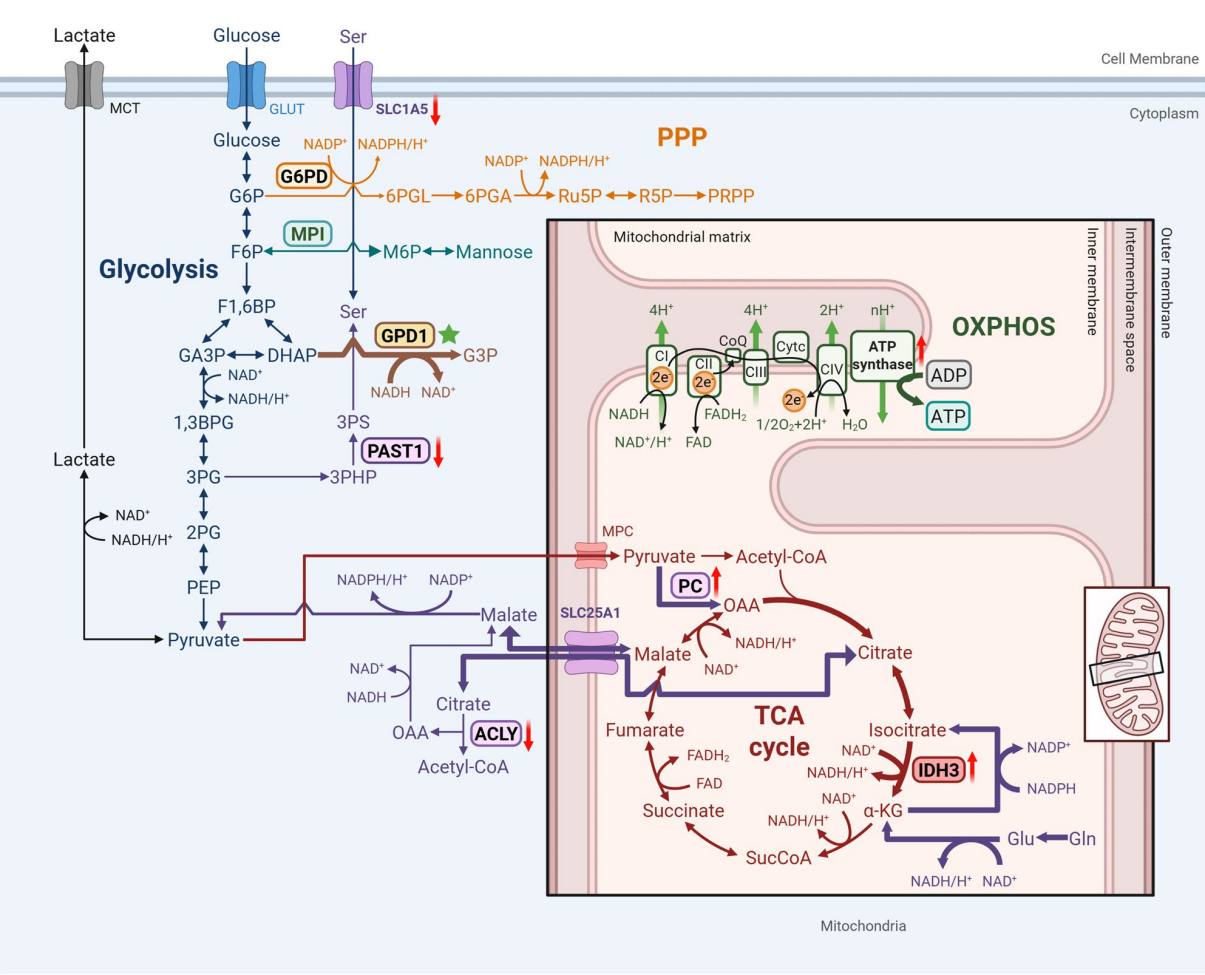
Glucose metabolism of dormant cancer cells and its interplay with other metabolic pathways. In most dormant cancer cells (DCCs), glucose metabolism is marked by attenuated glycolysis and enhanced oxidative phosphorylation (OXPHOS), driven by the upregulation of IDH3, SLC25A1, PC, and ATP synthase. By contrast, in luminal breast cancer, DCCs display reduced OXPHOS. The glucose metabolism of DCCs is tightly linked to lipid, amino acid, and nucleotide metabolism, with GPD1, ACLY, PSAT1, and G6PD serving as pivotal regulators. (Upward/downward arrows denote the upregulation/downregulation of key enzymes, while green stars indicate specific expression.) Abbreviations: GLUT, glucose transporter; G6P, glucose-6-phosphate; F6P, fructose-6-phosphate; F1,6BP, fructose 1–6 bisphosphate; GA3P, glyceraldehyde-3-phosphate; DHAP, dihydroxyacetone phosphate; NAD+, nicotinamide adenine dinucleotide; NADH, reduced nicotinamide adenine dinucleotide; 1,3BPG, 1,3-bisphosphoglycerate; 3PG, 3-phosphoglycerate; 2PG, 2-phosphoglycerate; PEP, phosphoenolpyruvate; MCT, monocarboxylate transporter; MPC, mitochondrial pyruvate carrier; OAA, oxaloacetate; IDH3, isocitrate dehydrogenase 3; α-KG, alpha-ketoglutarate; SucCoA, succinyl-CoA; FAD, flavine-adenine dinucleotide; FADH2, dihydroflavine-adenine dinucleotide; TCA, tricarboxylic acid; Gln, glutamine; Glu, glutamate; NADP+, nicotinamide adenine dinucleotide phosphate; NADPH, reduced nicotinamide adenine dinucleotide phosphate; CI, complex I; CII, complex II; CIII, complex III; Cytc, cytochrome c; CIV, complex IV; ADP, adenosine diphosphate; ATP, adenosine triphosphate; G6PD, glucose-6-phosphate dehydrogenase; 6PGL, 6-phosphogluconolactone; 6-PGA, 6-Phosphogluconate; Ru5P, ribulose 5-phosphate; R5P, ribose 5-phosphate; PRPP, phosphoribosyl pyrophosphate; PPP, pentose phosphate pathway; MPI, mannose phosphate isomerase; M6P, mannose-6-phosphate; 3PHP, 3-phosphohydroxypyruvate; PSAT1, phosphoserine aminotransferase 1; 3PS, 3-phosphoserine; Ser, serine; SLC1A5, serine transporter; PC, pyruvate carboxylase; SLC25A1, mitochondrial citrate carrier; GPD1, glycerol 3-phosphate dehydrogenase 1; G3P, glycerol-3-phosphate

The metabolic reprogramming of DCCs is tightly regulated by signaling pathways and transcriptional control. For instance, dormant melanoma cells express the proto-oncoprotein c-Myc, which preferentially occupies the promoters of OXPHOS genes (*IDH3A*, *IDH3B*, and *IDH3G*) to promote the expression of isocitrate dehydrogenase 3 (IDH3) [39]. IDH3 is a key enzyme in the tricarboxylic acid (TCA) cycle that catalyzes the conversion of isocitrate to alpha-ketoglutarate (α-KG) [44] (Fig. 2). Acting as an electron donor, α-KG regulates the oxygen sensor prolyl hydroxylase (PHD), which promotes hydroxylation and degradation of hypoxia-inducible factor-1 alpha (HIF-1α) [45], thereby inhibiting glycolysis and enhancing OXPHOS [39]. However, this regulatory mechanism is currently supported primarily by in vitro studies, and further in vivo validation is required. In dormant EOC cells, the upregulation of pyruvate carboxylase (PC) and SLC25A1, coupled with the downregulation of ATP citrate lyase (ACLY), the serine transporter SLC1A5, and phosphoserine aminotransferase 1 (PSAT1), leads to citrate replacing pyruvate as the primary carbon source for the TCA cycle [28]. Simultaneously, the upregulation of TCA cycle enzymes, including IDH2, succinate dehydrogenase complex subunit C (SDHC), and aconitase 2 (ACO2), accelerates the TCA cycle [28]. These alterations, in conjunction with upregulated ATP synthase, collectively enhance OXPHOS [28] (Fig. 2). Of note, these findings are largely derived from in vitro models and await in vivo confirmation.

Inhibition of glycolysis can induce cancer cell dormancy. In a study of brain-metastatic breast cancer cells, Sunderland et al. showed that inhibition of glycolysis activates the Hippo pathway, leading to phosphorylation of Yes-associated protein (YAP) [46]. As a transcriptional coactivator, YAP plays a central role in promoting cell proliferation, maintaining stem cell function, and preserving tissue homeostasis [47]. Once phosphorylated, YAP is prevented from translocating into the nucleus, thereby losing its ability to regulate downstream targets, which results in cell cycle arrest and induction of dormancy in breast cancer cells [46]. Moreover, Adverse microenvironmental factors, such as chemotherapy, hypoxia, acidosis, and nutrient deprivation, can induce cancer cell dormancy. These DCCs likewise exhibit the characteristic metabolic reprogramming of attenuated glycolysis and enhanced OXPHOS. In non-small-cell lung cancer (NSCLC), treatment with cisplatin or epidermal growth factor receptor (EGFR) inhibitors induces overexpression of the mitochondrial citrate carrier SLC25A1, driving cancer cells into dormancy and contributing to drug resistance [40]. SLC25A1, a monomeric member of the SLC25 family, operates through a ping-pong kinetic mechanism to mediate bidirectional exchange of citrate/isocitrate with malate [48]. Citrates participate in lipid synthesis in the cytoplasm and in the TCA cycle within mitochondria (Fig. 2). In DCCs, SLC25A1 transports citrate from the cytosol into the mitochondria, promoting OXPHOS to support cell survival [40].

A subset of DCCs displays distinctive metabolic features. In breast cancer, proliferative subtypes show divergent metabolic preferences: triple-negative and Her2⁺ breast cancer cells primarily depend on glycolysis for growth and proliferation, whereas luminal breast cancer cells mainly rely on mitochondrial respiration to maintain tumorigenic potential [49]. Consequently, their entry into and exit from dormancy involve distinct metabolic reprogramming.

In luminal breast cancer cells, OXPHOS is reduced during dormancy entry but enhanced during dormancy exit. Primary luminal breast cancer cells depend on estrogen receptor (ER) signaling to sustain self-renewal [31]. Hormone therapy (HT) suppresses ER expression, upregulates IL6R and CD133, and reduces mitochondrial activity, thereby inducing dormancy [31]. Persistent ER loss further promotes CD133^hi^ cells to secrete IL6 autonomously, which activates the IL6/STAT3/Notch3 pathway [31]. Activated Notch3 localizes to mitochondria, where it enhances OXPHOS, enabling DCCs to exit HT-induced dormancy, maintain self-renewal through an ER-independent mechanism, and simultaneously acquire drug resistance [31]. Interestingly, beyond IL6 autocrine signaling, dormant CD133^hi^ breast cancer cells can also exit dormancy through horizontal transfer of mitochondrial DNA (mtDNA) [50]. Cancer-associated fibroblasts (CAFs) in the TME encapsulate intact mitochondrial genomes into extracellular vesicles (EVs) [50]. Upon uptake by DCCs, this exogenous mtDNA boosts OXPHOS, facilitating dormancy exit, sustained self-renewal, and drug resistance [50].

### Lipid metabolism

Lipid metabolism consists of two major processes: lipid synthesis and lipid degradation. Key enzymes involved in lipid synthesis include ACLY, acetyl-CoA carboxylase (ACC), fatty acid synthase (FASN), acyl-CoA: diacylglycerol acyltransferase (DGAT), and acyl-CoA: cholesterol acyltransferase (ACAT), among others [51]. In contrast, lipid degradation is primarily mediated by enzymes such as carnitine palmitoyltransferase (CPT) and acyl-CoA dehydrogenase (ACAD) [51]. Importantly, acyl-CoA synthetase (ACS) plays a dual role, contributing to both lipid synthesis and lipid degradation [51]. By regulating the expression of these critical enzymes, DCCs reprograms their lipid metabolism.

**Fig. 3:**
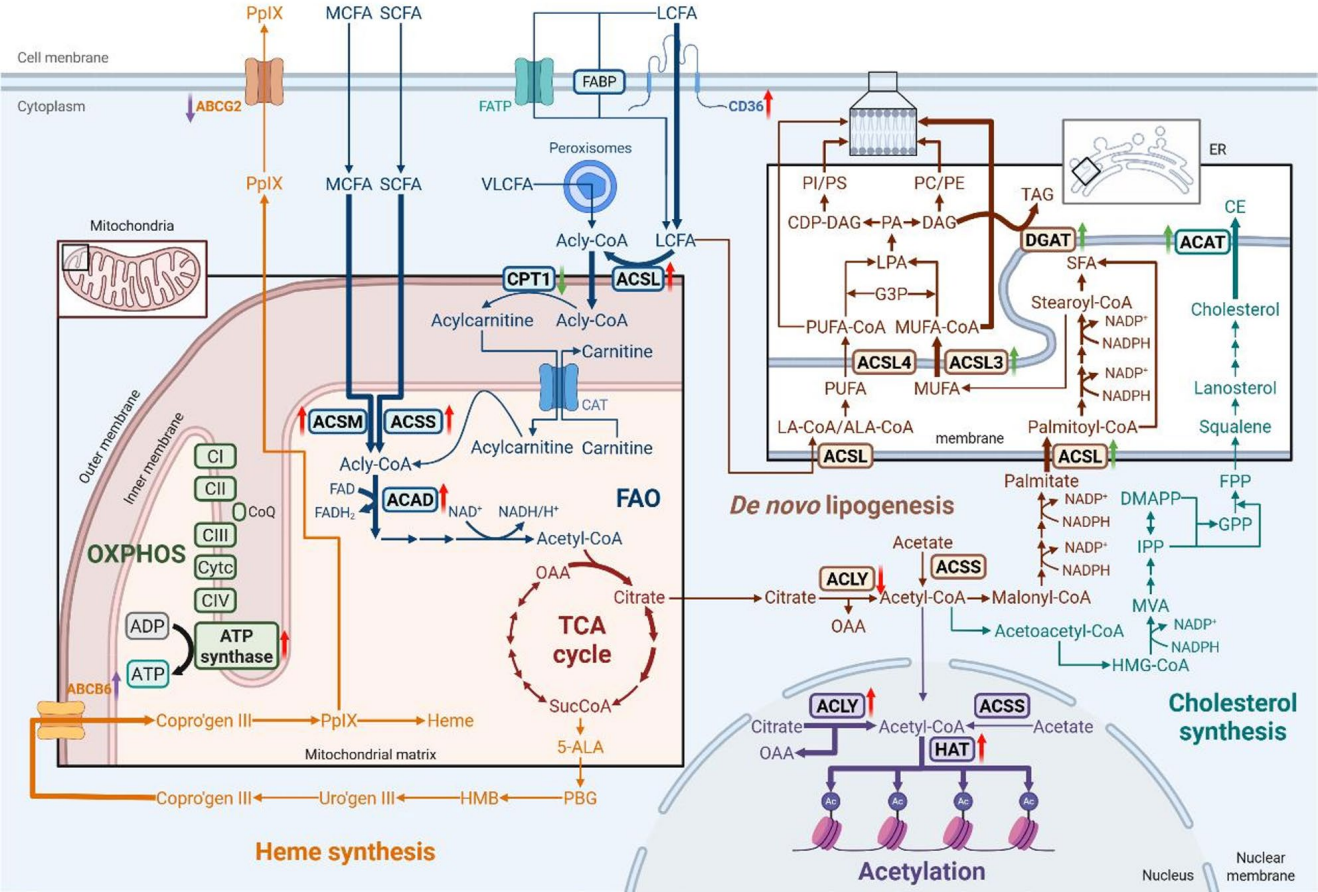
Lipid metabolism of dormant cancer cells and its interplay with other metabolic pathways. In most dormant cancer cells (DCCs), lipid metabolism is marked by enhanced fatty acid oxidation (FAO), driven by the upregulation of CD36, ACADVL, ACADM, HADH, ACSS2, and ACSM3. By contrast, a subset of DCCs displays enhanced lipid synthesis, mediated by the upregulation of ACSL3, DGAT1/2, and ACAT1, together with downregulation of CPT1. Lipid metabolism in DCCs is also interconnected with glucose and porphyrin metabolism, with GPD1, ACLY, ABCB6, and ABCG2 serving as key regulators. (Upward/downward arrows denote the upregulation/downregulation of key enzymes.) Abbreviations: VLCFA, very long chain fatty acids; LCFA, long chain fatty acids; MCFA, medium chain fatty acids; SCFA, short chain fatty acids; CD36, fatty acid transport protein; FABP, fatty acid binding protein; FATP, fatty acid transport protein; ACSL, acyl-CoA synthetase long chain family; CPT1, carnitine palmitoyltransferase 1; ACSM, acyl-CoA synthetase medium chain family; ACSS, acyl-CoA synthetase short chain family; ACADVL, very long chain acyl-CoA dehydrogenase; ACADM, medium chain acyl-CoA dehydrogenase; HADH, hydroxyacyl-CoA dehydrogenase; CAT, carnitine-acylcarnitine translocase; FAD, flavine-adenine dinucleotide; FADH_2_, dihydroflavine-adenine dinucleotide; NAD^+^, nicotinamide adenine dinucleotide; NADH, reduced nicotinamide adenine dinucleotide; OAA, oxaloacetate; SucCoA, succinyl-CoA; CI, complex I; CII, complex II; CIII, complex III; Cytc, cytochrome c; CIV, complex IV; ADP, adenosine diphosphate; ATP, adenosine triphosphate; 5-ALA, 5-aminolevulinic acid; PBG, porphobilinogen; HMB, hydroxymethylbilane; Uro’gen III, uroporphyrinogen III; Copro’gen III, coproporphyrinogen III; ABCB6/ABCG2, ATP-binding cassette transporter; PpIX, protoporphyrin IX; ACLY, ATP citrate lyase; SFA, saturated fatty acids; LA, linoleic acid; ALA, α-linolenic acid; PUFA, polyunsaturated fatty acid; MUFA, monounsaturated fatty acid; G3P, glycerol-3-phosphate; LPA, lysophosphatidic acid; PA, phosphatidic acid; DAG, diacylglycerol; CDP, cytidine diphosphate; PI, phosphatidylinositol; PS, phosphatidylserine; PC, phosphatidylcholine; PE, phosphatidylethanolamine; DGAT, acyl-CoA: diacylglycerol acyltransferase; TAG, triacylglycerol; HMG-CoA, 3-hydroxy-3-methylglutaryl-CoA; MVA, mevalonic acid; IPP, isopentenyl diphosphate; DMAPP, dimethylallyl diphosphate; GPP, geranyl pyrophosphate; FPP, farnesyl pyrophosphate; ACAT, acyl-CoA: cholesterol acyltransferase 1; CE, cholesterol ester; ER, endoplasmic reticulum; HAT, histone acetyltransferase

Enhanced fatty acid oxidation (FAO) is a defining feature of lipid metabolism in DCCs. Dormant oral squamous cell carcinoma (OSCC) cells overexpress the fatty acid transporter CD36 and lipid metabolic enzymes, thereby facilitating fatty acids (FAs) uptake and β-oxidation [27] (Fig. 3). Moreover, exposure to exogenous FAs acts on CD36 in a dose-dependent manner, augmenting the metastatic potential of DCCs [27]. CD36-mediated FAs uptake involves a finely tuned regulatory mechanism. In gastric cancer (GC) cells, cysteine residues (Cys333 and Cys272) within CD36 form a disulfide bond that stabilizes the N-terminal conformation between helical and sheet structures, enabling the extracellular domain of CD36 to cover the binding pocket of long-chain fatty acids (LCFAs) and thus prevent their transport into the cytoplasm [52]. Hydrogen sulfide (H₂S) promotes CD36 overexpression, induces nuclear translocation of nuclear factor erythroid 2-related factor 2 (NRF2), and disrupts the Cys333–Cys272 disulfide bond, thereby switching CD36 to its LCFA-binding conformation, ultimately enhancing LCFAs uptake [52].

Enhanced FAO is not only critical for maintaining dormancy but also indispensable for its induction. ACLY catalyzes the cleavage of citrate into oxaloacetate and acetyl-CoA, with the latter serving as a substrate for protein acetylation [53] (Fig. 3). Following serum deprivation in vitro, colorectal cancer (CRC) cells display enhanced FAO, which promotes nuclear ACLY expression and increases acetyl-CoA production [7]. p300, a histone acetyltransferase (HAT) with homology and functional synergy to CREB-binding protein (CBP), further amplifies this process [54]. Overexpression of CBP/p300 induces acetylation of histone H3 at lysine 27 (H3K27Ac) at the *Nanog* promoter, thereby activating *Nanog* transcription [7]. Nanog, a master transcription factor essential for maintaining stem cell self-renewal and pluripotency [55], subsequently upregulates COUP-TF1, DEC2, p21, and p27, ultimately inducing dormancy in CRC cells [7]. Nevertheless, this regulatory mechanism is primarily based on in vitro studies, and in vivo evidence remains limited at present. Beyond serum deprivation, certain FAs can also trigger dormancy in CRC cells. For example, under the catalysis of 15-lipoxygenase-1 (LOX15), linoleic acid (LA) metabolites activate peroxisome proliferator-activated receptor-gamma (PPAR-γ), thereby inhibiting proliferation and inducing apoptosis in cancer cells [56]. Further studies demonstrated that LA induces mitochondrial oxidative stress, leading to the upregulation of microRNA-494 (miR-494), which suppresses the expression of target genes *MYCC* and *PGC1α*, ultimately driving CRC cells into dormancy [57]. It should be noted that these findings remain confined to in vitro studies and require further in vivo validation.

Enhanced lipogenesis represents a characteristic feature of lipid metabolism in certain DCCs, underscoring the heterogeneity of lipid metabolism. In salivary adenoid cystic carcinoma (SACC), miR-922 functions as a dormancy-associated microRNA (DmiR) [32]. It is highly expressed in proliferating SACC cells and directly targets the 3′ untranslated region (3′UTR) of DEC2 mRNA, thereby suppressing DEC2 expression [32]. Further studies revealed that a HIF-1α/miR-922/DEC2 feedback loop mediates hypoxia-induced dormancy and lipid metabolic reprogramming in SACC cells [32]. Under hypoxic conditions, HIF-1α downregulates miR-922, which in turn upregulates DEC2^32^. Elevated DEC2 enhances the expression of key enzymes involved in triglyceride synthesis (DGAT1/2) and cholesterol ester synthesis (ACAT1), while repressing the expression of CPT1, a rate-limiting enzyme in FAO [32]. These changes lead to the accumulation of free fatty acids (FFAs) and triglycerides (TGs) in blood lipids [32] (Fig. 3).

In DCCs, enhanced lipogenesis inhibits ferroptosis and promotes metastasis. DCCs overexpress ACS, which enhances lipid metabolism [58]. Functioning downstream of FASN, ACS converts FAs into acyl-CoA, a pivotal intermediate in lipid metabolic pathways including phospholipid biosynthesis, protein lipidation, and β-oxidation [59]. Among the five human long-chain acyl-CoA synthetase family members (ACSL1, 3–6), ACSL4 preferentially activates polyunsaturated fatty acids (PUFAs), whereas ACSL3 favors monounsaturated fatty acids (MUFAs) [60]. PUFAs promote ferroptosis by inducing lipid peroxidation, while MUFAs inhibit this process [61]. Dormant luminal breast cancer cells overexpress ACSL3, thereby enhancing de novo lipogenesis [62]. The resulting MUFAs are incorporated into cell membranes, where they suppress lipid peroxide accumulation, protecting DCCs from ferroptosis and facilitating metastasis [62].

In DCCs, lipid metabolism is interconnected with glucose and porphyrin metabolism. Studies on acute myeloid leukemia have confirmed that, following chemotherapy, leukemic stem cells rely on FAO-driven OXPHOS for survival^63^,^64^. Whether enhanced FAO promotes OXPHOS in DCCs, however, remains to be experimentally validated. What is certain is that, within DCCs, enhanced lipogenesis correlates with diminished glycolysis. Dormant glioblastoma (GBM) cells uniquely express glycerol-3-phosphate dehydrogenase 1 (GPD1), which is absent in proliferating cancer cells^65^. Acting as a critical bridge between glucose and lipid metabolism, GPD1 catalyzes the conversion of reduced nicotinamide adenine dinucleotide (NADH) and dihydroxyacetone phosphate (DHAP) into NAD⁺ and glycerol-3-phosphate (G3P) 66, thereby suppressing glycolysis while promoting lipid synthesis. Yet, the regulation of GBM dormancy metabolism by GPD1 remains largely confined to in vitro studies, and its in vivo relevance warrants further investigation. In addition, enhanced lipid metabolism further upregulates the peptide transporter PEPT1 and the ATP-binding cassette transporter ABCB6, while downregulating ABCG2, collectively driving porphyrin metabolism and protoporphyrin IX (PpIX) accumulation in DCCs^58^ (Fig. 3).

### Amino acid metabolism

Amino acids serve as essential energy sources for cancer cell proliferation, particularly glutamine, asparagine, arginine, methionine, serine, and cysteine [67]. Although amino acid anabolism is highly active in cancer cells, it remains insufficient to meet their metabolic demands [68]. Consequently, proliferating cancer cells must continuously acquire large amounts of amino acids from the extracellular environment [67, 68]. Notably, deprivation of specific amino acids can induce autophagy and dormancy in cancer cells, and this amino acid starvation-induced autophagy is crucial for the survival of DCCs [69]. A representative example is dormant EOC cells, which rely on autophagy to survive under nutrient-deficient conditions [28]. PSAT1 catalyzes the conversion of 3-phosphohydroxypyruvate (3PHP) into 3-phosphoserine (3PS), functioning as a critical metabolic bridge linking glucose and amino acid metabolism [70]. Compared with proliferating EOC cells, DCCs downregulate SLC1A5 and PSAT1, resulting in decreased serine uptake and biosynthesis [28] (Fig. 2). Interestingly, while inhibition of serine uptake or metabolism may impair the ability of primary EOC tumors to form spheroids, serine deficiency does not affect the recurrence or metastatic potential of DCCs, likely due to compensatory amino acid starvation-induced autophagy [28]. As noted above, these findings are primarily derived from in vitro models and await further in vivo validation.

Amino acid starvation-induced autophagy depends on the formation of the autophagosome initiation complex (AIC). DIRAS family GTPase 3 (*DIRAS3*) is a maternally imprinted tumor suppressor gene encoding a 26 kDa GTPase that plays a central role in tumor suppression, autophagy, and dormancy [71]. Under normal conditions, DIRAS3 is downregulated in EOC cells, which allows B-cell lymphoma 2 (BCL2) to bind Beclin 1 (BECN1) homodimers and thereby prevents assembly of the autophagosome initiation complex (AIC) [72]. This complex consists of DIRAS3, BECN1, class III phosphatidylinositol-3-kinase (PI3KC3), autophagy-related 14 (ATG14), and phosphoinositide-3-kinase regulatory subunit 4 (PI3KR4) [72]. When nutrients, particularly amino acids, are scarce, DCCs overexpress DIRAS3. The GTP-binding central domain of DIRAS3 interacts with the N-terminus of BECN1, disrupting BECN1 homodimers and preventing BCL2 from binding to BECN1, which leads to the formation of the DIRAS3–BECN1 heterodimer [72]. DIRAS3 further promotes the association of BECN1 monomers with PIK3C3 and ATG14, enhances PIK3C3 activity, and facilitates AIC assembly, thereby inducing autophagy and ultimately promoting DCCs survival [72]. In addition to DIRAS3, insulin-like growth factor 2 (IGF2) can also induce autophagy under nutrient deprivation[73]. Chemotherapy-induced dormant osteosarcoma cells overexpress IGF2 and rely on both autophagy and glutamine metabolism to maintain their survival[73]. However, these autophagic regulatory mechanisms are largely based on in vitro studies, and in vivo evidence is currently limited.

Although tryptophan cannot serve as an energy source, its metabolites play important roles in signal transduction [74]. The majority of tryptophan is catabolized via the kynurenine (Kyn) pathway to generate Kyn. This step is catalyzed by three rate-limiting enzymes: indoleamine 2,3-dioxygenase 1 (IDO1), IDO2, and tryptophan 2,3-dioxygenase 2 (TDO2), which are distributed in different tissues. IDO1 is expressed in multiple tissues, IDO2 is primarily expressed in the immune system, and TDO2 is mainly expressed in the liver [75]. Kyn binds to and activates the aryl hydrocarbon receptor (AhR). Subsequently, the activated AhR forms a heterodimer with the AhR nuclear translocator (ARNT) and translocates into the nucleus, where it regulates the transcription of specific target genes [76].

The Kyn/AhR pathway plays a vital role across the induction, maintenance, and reawakening phases of dormancy. In androgen-dependent prostate cancer cells, the androgen receptor (AR) suppresses glucocorticoid receptor (GR) expression and inhibits *TDO2* transcription by binding to an intronic region of *TDO2*^77^. Concurrently, EZH2-mediated H3K27me3 on the *TDO2*-associated transposon (L1PA5) blocks GR binding, thereby repressing GR-driven transcriptional activity of *TDO2* [77]. Although androgen deprivation therapy (ADT) is the standard treatment for androgen-sensitive prostate cancer, most patients eventually relapse [78], which is closely associated with ADT-induced DCCs. During dormancy induction, ADT upregulates p27, resulting in cell cycle arrest and the formation of DCCs [77]. In the dormancy maintenance phase, ADT downregulates AR and upregulates GR, forming a transcriptional loop between L1PA5 and the *TDO2* promoter to enhance the transcription of *TDO2*. Overexpression of TDO2 sustains the survival of DCCs via the Kyn/AhR pathway [77]. During dormancy reawakening, persistent GR-driven *TDO2* expression reactivates DCCs through the Kyn/AhR pathway, conferring castration resistance [77].

Inhibition of the Kyn/AhR pathway decreases Glucose-6-phosphate dehydrogenase (G6PD) activity, disrupts redox homeostasis, and promotes the apoptosis of DCCs. Glucose-6-phosphate dehydrogenase (G6PD), the rate-limiting enzyme of the PPP, generates nicotinamide adenine dinucleotide phosphate (NADPH), which maintains intracellular redox balance by reducing oxidized glutathione (GSSG) to its reduced form (GSH) [79]. In melanoma cells, interferon-beta (IFN-β) not only directly upregulates p27, but also suppresses Src kinase expression via the IDO1/Kyn/AhR pathway, the latter resulting in a reduction of tyrosine-phosphorylated STAT3 [p-STAT3(Y)] [80]. Subsequently, serine-phosphorylated STAT3 [p-STAT3(S)] translocates into the nucleus, promotes the expression of p27, and induces cancer cell dormancy [80]. Inhibition of AhR prevents its nuclear translocation, thereby relieving the suppression of Src kinase expression. This leads to increased p-STAT3(Y) and facilitates the nuclear translocation of p-STAT3(S, Y), which in turn enhances the expression of p53 [81]. p53 binds to and inhibits G6PD activity, disrupts redox homeostasis, and ultimately promotes the apoptosis of DCCs [81].

In DCCs, amino acid metabolism is characterized by the utilization of autophagy to supply energy when energy-supplying amino acids are deficient. Moreover, the tryptophan metabolite Kyn regulates the expression of specific genes through the Kyn/AhR signaling axis, playing pivotal roles in inducing, maintaining, and reawakening dormancy, as well as in sustaining redox homeostasis. Current knowledge of amino acid metabolism in DCCs may represent only the tip of the iceberg, and future studies are expected to fully elucidate its mechanisms.

### Nucleotide metabolism

Proliferating cancer cells synthesize large quantities of nucleotide triphosphates (NTPs) and their deoxy counterparts (dNTPs) to sustain continuous cell cycle progression [82]. For DCCs, although close associations between nucleotide synthesis and cancer cell dormancy are implicated, definitive nucleotide metabolic characteristics of DCCs have yet to be established.

Inhibition of nucleotide synthesis can induce cancer cell dormancy. For instance, mannose can suppress cancer cell proliferation, particularly in cells that lack or express low levels of mannose phosphate isomerase (MPI) [83]. Derived from the glycolytic intermediate fructose-6-phosphate (F-6-P), mannose is metabolized by MPI, which catalyzes the interconversion of F-6-P and mannose-6-phosphate (M-6-P) [84] (Fig. 2). Yoichiro et al. demonstrated that in *MPI*-knockout human cancer cells, mannose treatment reduces glycolysis, increases reliance on OXPHOS, and decreases utilization of phosphoribosyl pyrophosphate (PRPP), thereby suppressing de novo dNTP synthesis [29]. PRPP functions as an essential ribose donor for purine and pyrimidine biosynthesis and is also required for the synthesis of NAD, histidine, and tryptophan [85]. Consequently, dNTP deficiency impedes DNA replication, thereby contributing to cell cycle arrest and dormancy induction [29]. Nonetheless, these findings remain confined to in vitro studies and require further in vivo validation.

Conversely, increased nucleotide synthesis serves as a crucial marker for reactivation of DCCs. In dormant Her2⁺ breast cancer cells, glycolysis is suppressed while OXPHOS and FAO are enhanced. These metabolic shifts elevate ROS levels, leading to compensatory upregulation of NRF2 to maintain redox homeostasis [86]. NRF2 is a master regulator of antioxidant responses and a driver of cancer therapy resistance [87]. Beyond its canonical role in redox regulation, NRF2 also promotes de novo nucleotide synthesis by upregulating 6-phosphogluconate dehydrogenase (6PGD), the key enzyme of PPP, thereby facilitating the reactivation of DCCs and subsequent tumor recurrence [86].

To date, diminished nucleotide synthesis has only been identified as a hallmark of DCCs in Her2⁺ breast cancer. Whether this metabolic feature is present in DCCs across other cancer types remains to be experimentally validated.

## Treatment plans targeting the metabolic pathways of dormant cancer cells

Surgical resection, radiotherapy, and chemotherapy remain the cornerstone treatments in clinical oncology, but recurrence after prolonged disease-free survival continues to pose a major challenge [88]. Although emerging approaches such as targeted therapy and immunotherapy have achieved durable responses in certain cancers [89, 90], their efficacy is limited, as not all patients benefit and drug resistance frequently develops [91, 92]. DCCs are increasingly recognized as a principal driver of recurrence and therapy resistance, and the absence of safe and effective strategies to specifically target them represents a leading cause of mortality in patients with advanced disease and multidrug resistance [93].

Integrated analyses of metabolic pathways in DCCs have revealed convergent patterns of metabolic reprogramming across diverse tumor types. Consequently, inhibiting critical nodes within these hallmark pathways offers opportunities for precision therapy. Moreover, in some DCCs, multiple metabolic pathways form interactive networks, suggesting that targeting key cross-regulatory hubs could simultaneously disrupt several pathways and thereby enhance therapeutic efficacy. In this section, we highlight both approaches and discuss therapeutic strategies designed to target the metabolic vulnerabilities of DCCs.

### Treatment plans targeting glucose metabolism

Reduced glycolysis and increased OXPHOS are characteristic metabolic features of many DCCs. Consequently, inhibition of OXPHOS has emerged as a promising therapeutic strategy. Several agents currently under preclinical investigation, with potential for clinical application, include IACS-01075974 [94], UCN-01 (7-hydroxystaurosporine) [95], ESI-09 [96], and citrate transport protein inhibitor-2 (CTPI-2) [40] (Table 1).

**Table 1:** Inhibitors for targeting oxidative phosphorylation in dormant cancer cells

Name	Type	Mechanism of Action	Tested Cancers	Research Status
IACS-010759	Mitochondrial complex I inhibitor	It binds to the ND1 subunit at the entrance of the ubiquinone channel, thereby inhibiting Complex I [97].	Solid tumors, AML	A phase I trial for advanced solid tumors and AML showed modest efficacy and narrow therapeutic index of IACS-010759 [98].
UCN-01	Serine/threonine kinase inhibitor; mitochondrial uncoupler	It inhibits Chk1 and cTAK1, abolishing the G2/M DNA damage checkpoint, thereby inducing mitochondria-dependent apoptosis; also inhibits CDK2, PDK1, multiple kinases including MARK3, PKC, GSK3β, CDK1, and Chk2, exhibiting broad biological effects[99, 100].	Ovarian cancer, TNBC, SCLC, pancreatic cancer	Four phase I trials in solid tumors demonstrated good tolerability [101–104], and phase II trials have been initiated to evaluate its efficacy in ovarian cancer [105], TNBC [106], SCLC and pancreatic cancer. However, no significant antitumor activity was observed in ovarian cancer or TNBC [105, 106]. In contrast, three phase I trials for solid tumors and/or lymphomas showed a lack of efficacy and ≥grade 3 adverse events [107–109].
ESI-09	Mitochondrial uncoupler; EPAC inhibitor	It acts as a proton carrier, disrupting the proton gradient across the mitochondrial inner membrane [96].	NSCLC	Currently in preclinical studies, ESI-09 exhibits good antitumor activity under low-glucose conditions, shows a better safety profile compared to conventional chemotherapy [96].
CTPI-2	SLC25A1 Inhibitor	It inhibits the transport of citrate from the cytoplasm into mitochondria, thereby suppressing the TCA cycle and OXPHOS [40].	NSCLC	Currently in preclinical studies, CTPI-2 shows enhanced antitumor activity when combined with cisplatin or EGFR inhibitors [40].

*Abbreviations*: *AML* acute myeloid leukemia, *Chk1* checkpoint kinase 1, *cTAK1* Cdc25C-associated protein kinase 1, *CDK2* cyclin-dependent kinase 2, *PDK1* phosphoinositide-dependent kinase 1, *MARK3* multiple kinases including microtubule affinity regulating kinase 3, *PKC* protein kinase C, *GSK3β* glycogen synthase kinase 3beta, *CDK1* cyclin-dependent kinase 1, *Chk2* checkpoint kinase 2, *TNBC* triple-negative breast cancer, *SCLC* small cell lung cancer, *NSCLC* non-small cell lung cancer

IACS-010759 is a novel mitochondrial complex I inhibitor that binds specifically to the ND1 subunit, blocking electron transport chain activity, suppressing OXPHOS, and reducing ATP generation, thereby inducing an energy crisis in tumor cells [110]. Additionally, IACS-010759 promotes metabolic reprogramming, forcing cancer cells to rely on glycolysis and thereby enhancing their sensitivity to radiotherapy and chemotherapy [111]. Current evidence indicates that its anticancer effects are most pronounced in OXPHOS-dependent tumor populations, including both intrinsically OXPHOS-dependent cells and those rendered OXPHOS-dependent by adverse microenvironments. The latter group shares metabolic traits with DCCs, although the term “dormant cancer cells” has not been explicitly applied in the literature. Preclinical studies have demonstrated that IACS-010759 directly inhibits OXPHOS in inherently OXPHOS-dependent cancers, such as neuroblastoma and SWI/SNF-mutant lung cancer, leading to apoptosis due to metabolic inflexibility [112, 113]. It has also been shown to reverse drug resistance and suppress tumor recurrence in models of drug-resistant ovarian cancer and EGFR inhibitor-resistant NSCLC by targeting mitochondrial metabolism [114, 115]. Compared with other OXPHOS inhibitors, including metformin, atovaquone, and BAY 87-2243, IACS-010759 offers greater precision in targeting metabolically fragile tumors and reversing resistant phenotypes via inhibition of metabolic remodeling [116].

However, the clinical development of IACS-010759 has been hampered by systemic toxicity and insufficient tumor selectivity [94]. Two dose-escalation phase I trials (NCT02882321, NCT03291938) were terminated early due to dose-limiting toxicities, including elevated blood lactate and neurotoxicity, resulting from the narrow therapeutic index of IACS-010759 [98].

UCN-01, originally identified as a protein kinase C (PKC) inhibitor, was later found to be a broad serine/threonine kinase inhibitor with diverse biological activities [100]. UCN-01 inhibits checkpoint kinase 1 (Chk1), abrogates the G2/M DNA damage checkpoint, forces cells with unrepaired DNA damage into mitosis, and triggers mitochondria-dependent apoptosis via mitochondrial membrane potential loss, cytochrome c release, and caspase activation [99]. Kondoh et al. demonstrated that UCN-01 suppresses and kills dormant EOC cells in vitro [117]. As noted earlier, dormant EOC cells express mitochondrial metabolism-related genes and depend on OXPHOS for survival [28]. In vitro, UCN-01 outperformed cisplatin and paclitaxel in suppressing DCCs and showed synergy with the mitochondrial inhibitor oligomycin [95]. In vivo, UCN-01 delayed tumor recurrence when administered as maintenance therapy after carboplatin treatment, whereas continued carboplatin led to rapid relapse [95].

However, the lack of kinase specificity of UCN-01 reduces its therapeutic efficacy due to off-target effects. Several phase I trials combining UCN-01 with conventional chemotherapy have been conducted. Among them, three phase I trials—UCN-01 in combination with topotecan in ovarian cancer^101^, with irinotecan in triple-negative breast cancer (TNBC) [102], and with carboplatin in small cell lung cancer (SCLC) [104]—demonstrated favorable safety and tolerability. Unfortunately, two phase II trials in ovarian cancer and TNBC failed to show significant antitumor activity, ultimately leading to unsuccessful outcomes [105, 106].

Collectively, the clinical experience with IACS-010759 and UCN-01 highlights a recurring challenge in targeting OXPHOS: achieving sufficient antitumor activity while maintaining an acceptable safety. These lessons underscore the need for tumor-selective delivery strategies and rational combination regimens that exploit metabolic vulnerabilities without exacerbating systemic toxicity, thereby widening the therapeutic window.

ESI-09, initially characterized as an exchange protein directly activated by cAMP (EPAC) inhibitor, has been studied in cancer [118] and neurological diseases [119]. Unexpectedly, Maeda et al. found that ESI-09 shares structural similarity with the classical mitochondrial uncoupler FCCP, functioning as a proton carrier that dissipates the mitochondrial proton gradient, decouples electron transport from ATP synthesis, and accelerates ATP and substrate depletion, thereby inhibiting OXPHOS independently of EPAC inhibition [96]. Notably, ESI-09 demonstrated superior safety and persistence compared with FCCP and did not induce mitochondrial membrane depolarization [96]. In a low-glucose, acidic microenvironment—conditions that promote reduced glycolysis and enhanced OXPHOS in lung cancer cells—ESI-09 was markedly more cytotoxic against DCCs than cisplatin in vitro [96]. In vivo, ESI-09 effectively inhibited tumor growth without synergistic toxicity when combined with bevacizumab [96]. However, this study only assessed the short-term effects of ESI-09 on DCCs induced by a low-glucose acidic microenvironment. Its efficacy in other types of DCCs or under different tumor microenvironments remains to be determined. Moreover, the potential side effects of EPAC inhibition and the safety of long-term use require further investigation.

CTPI-2 is a recently developed SLC25A1 inhibitor with greater affinity and intrinsic activity than earlier inhibitors such as benzenetricarboxylate (BTA) and CTPI-1 [40]. Because the production of NADH and FADH2 within the TCA cycle directly influences OXPHOS efficiency [120], citrate transport plays a critical regulatory role. Citrate serves as a negative allosteric regulator of both phosphofructokinase 1 (PFK1) in glycolysis and citrate synthase in the TCA cycle [121]. CTPI-2 increases cytoplasmic citrate levels by preventing its mitochondrial transport, and the accumulated cytoplasmic citrate in turn suppresses PFK1 activity, thereby reducing both glycolysis and OXPHOS and lowering the risk of tumor recurrence [40]. Importantly, CTPI-2 exhibited greater anticancer activity in combination with platinum agents or the EGFR inhibitor AZD9291 compared with monotherapy [40]. These findings suggest that CTPI-2 may represent a promising therapeutic option for resistant NSCLC. Although dormant EOC cells also overexpress SLC25A1, it remains uncertain whether CTPI-2 could be applied in this context.

### Treatment plans targeting lipid metabolism

In most DCCs, alterations in lipid metabolism are primarily characterized by enhanced FAO. A key driver of this phenotype is the overexpression of the fatty acid receptor CD36 [27, 52]. Inhibition of CD36 not only blocks FAs uptake and lipid metabolic reprogramming—leading to DCC death through impaired energy production and biosynthesis—but also alleviates the immunosuppressive effects of regulatory T cells (Tregs), thereby enhancing CD8⁺ T-cell cytotoxicity, remodeling the tumor immune microenvironment, and suppressing metastatic recurrence [122]. Recent studies have identified several classes of compounds targeting CD36 in cancer cells, including small-molecule antagonists, peptides, natural proteins, and neutralizing antibodies [123] (Table 2). For example, Tzeng et al. developed a humanized anti-CD36 IgG4 antibody (PLT012) that targets the lipid-binding domain of CD36 and demonstrated excellent safety and favorable pharmacokinetics in mice and cynomolgus macaques [124]. PLT012, either as monotherapy or in combination with PD-L1 blockade or standard immunotherapy, elicited robust antitumor immunity in both immunotherapy-sensitive and -resistant hepatocellular carcinoma (HCC) models124. Similarly, Takaichi et al. reported that the small-molecule sulfosuccinimidyl oleate (SSO) significantly inhibited CD36-overexpressing OSCC cells, and they proposed a therapeutic strategy combining CD36 inhibitors with immune checkpoint inhibitors to exploit the immunomodulatory effects of CD36 inhibition [125].

**Table 2:** Compounds for targeting CD36 in cancer cells

Type	Compound	Mechanism of Action	Tested Cancers	Research Status
Small molecule	SSO	It irreversibly binds to CD36, inhibiting the uptake of FAs and oxidized oxLDL [125].	OSCC	Currently in preclinical studies, it inhibited OSCC growth and promoted antitumor immune responses [125].
Peptide	VT1021	It induces MDSCs to express TSP-1, which mediates apoptosis and remodels TIME by binding to CD36 and CD47 [126].	Ovarian cancer, pancreatic cancer, TNBC, GBM	A phase I trial in ovarian cancer, pancreatic cancer, TNBC, and GBM demonstrated good safety and tolerability^126^. A Phase II/III trial is investigating efficacy in GBM.
Natural protein	Vstat120	It binds to the CLESH domain of CD36, inhibiting endothelial cell migration and angiogenesis [127].	GBM	Currently in preclinical studies, although it suppresses GBM growth, further research is needed to identify the minimal functional regions responsible for its antitumor effects [127].
Antibody	JC63-1	It blocks FAs and oxLDL uptake [27].	OSCC	Currently in preclinical studies, it almost completely inhibited OSCC metastasis without causing observable side effects [27].
	PLT012	It blocks CD36-mediated lipid metabolic reprogramming and reshapes the TIME [124].	Primary HCC, liver metastases	Currently in preclinical studies, it demonstrated excellent safety, favorable pharmacokinetics, and strong antitumor immunity against primary HCC and liver metastases [124].
Nanoparticles (NPs)	HA@CD36i -TR@siSCD1	HA gel (shell) releases drugs in response to acidic TME and hyaluronidase; TR cationic micelle (core) release drugs upon exposure to GSH; SSO (CD36i) suppresses exogenous FAs uptake; siSCD1 inhibits endogenous FAs synthesis [128].	Prostate cancer	Currently in preclinical studies, HA-TR nanoparticle system possesses strong tumor-targeting and penetration capabilities. HA@CD36i-TR@siSCD1 exhibited significant synergistic effects, effectively suppressing prostate cancer growth, invasion, and metastasis [128].
	LA-Cys, hemin and SSO three-in-one self-amplifying nanodrug (LHS NPs)	LA-Cys inactivates GPX4 and depletes intracellular GSH, thereby inducing canonical ferroptosis; Hemin converts endogenous H₂O₂ into ·OH, triggering noncanonical ferroptosis; SSO inhibits CD36-mediated FAs uptake [129].	TNBC	Currently in preclinical studies, it showed potent efficacy by inhibiting TNBC growth, suppressing lung metastases, and prolonging overall survival [129].
Others	niraparib	It promotes nuclear translocation of NRF2, upregulates CD36 expression, thereby enhancing FAs uptake, ultimately inducing ferroptosis [130].	Ovarian cancer	It was approved by the FDA in March 2017 as a PARP inhibitor for the treatment of ovarian, fallopian tube, and primary peritoneal cancers. Recent preclinical studies revealed that it limits peritoneal metastasis in ovarian cancer by inducing CD36-dependent ferroptosis [130].

*Abbreviations*: *SSO* sulfosuccinimidyl oleate, *FAs* fatty acids, *oxLDL* oxidized low-density lipoproteins, *OSCC* oral squamous cell carcinoma, *MDSCs* myeloid-derived suppressor cells, *TSP-1* thrombospondin-1, *TIME* tumor immune microenvironment, *TNBC* triple-negative breast cancer, *GBM* glioblastoma, *Vstat120* vasculostatin, *HCC* hepatocellular carcinoma, *HA* hyaluronic acid, *TME* tumor microenvironment, *TR* tumor-targeting, *GSH* glutathione, *siSCD1* stearoyl-CoA desaturase 1 siRNA, *GPX4* glutathione peroxidase 4, *H*_*2*_*O*_*2*_ hydrogen peroxide, *OH* hydroxyl radicals, *LA-Cys* Linoleic acid-cysteamine, *NRF2* nuclear factor erythroid 2-related factor 2, *PARP* poly (adenosine diphosphate-ribose) polymerase

Beyond CD36, upregulation of lipid metabolic enzymes also contributes to enhanced FAO in DCCs. For instance, dormant prostate cancer cells overexpress acyl-CoA synthetase short-chain family member 2 (ACSS2) and medium-chain family member 3 (ACSM3) [58], while dormant OSCC cells overexpress very-long-chain acyl-CoA dehydrogenase (ACADVL), medium-chain acyl-CoA dehydrogenase (ACADM), and hydroxyacyl-CoA dehydrogenase (HADH), all of which promote FAO [27]. Unfortunately, research on these enzymes remains limited, and the mechanisms driving their upregulation are poorly understood. Moreover, the lack of safe and effective inhibitors currently hinders their clinical application.

Distinct from this pattern, some DCCs exhibit enhanced lipid synthesis [32, 62]. In luminal breast cancer, enhanced lipogenesis protects DCCs from ferroptosis and facilitates metastasis [62]. In line with this, Puente-Cobacho et al. demonstrated that treatment of dormant breast cancer cells with Fasnall (a FASN inhibitor) markedly reduced viability of DCCs, whereas SSO (a CD36 inhibitor) had little effect [62], likely reflecting the limited capacity of these cells for β-oxidation of endogenous FAs. Thus, FASN inhibitors represent a promising strategy to induce ferroptosis and suppress metastasis in dormant luminal breast cancer.

However, dormant breast cancer cells can also evade ferroptosis by secreting Dickkopf-1 (DKK1), a secreted protein originally implicated in embryonic head development [131, 132]. Therefore, single-agent therapy using a FASN inhibitor may not yield optimal therapeutic outcomes. The Wnt/β-catenin pathway can promote *DKK1* transcription, and the secreted DKK1 subsequently binds to the Wnt receptor LRP5/6, thereby blocking Wnt/β-catenin signaling and reducing STAT3 activation [131]. Under normal conditions, STAT3 represses expression of SLC7A11, a component of the cystine/glutamate antiporter system Xc⁻ that plays a key role in ferroptosis [131, 133]. By suppressing STAT3, DKK1 indirectly promotes SLC7A11 expression, elevates intracellular GSH, reduces lipid peroxidation, and protects DCCs from ferroptosis, ultimately promoting metastasis [131]. Notably, DCCs can acquire durable resistance to Erastin-induced ferroptosis, potentially through Erastin-mediated upregulation of DKK1; combining DKK1 inhibitors with Erastin markedly improves therapeutic efficacy [131]. Although FASN inhibitors act via mechanisms distinct from classical ferroptosis inducers (such as Erastin, RSL3, or iFSP1), it remains unclear whether FASN inhibition might also upregulate DKK1 and thereby induce resistance. Experimental studies are needed to determine whether combination therapy with FASN inhibitors and DKK1 blockade provides superior efficacy over FASN inhibition alone in dormant breast cancer.

In dormant GBM cells, enhanced lipid synthesis is driven by the specific expression of GPD1 [65]. Several GPD1 inhibitors, including methylarsenite [134], iGP-1/5 (ChemBridge: 5224148/5224147) [135], and α-tocopheryl succinate (TOS) [136], have been reported; however, these remain at the experimental stage and have not yet advanced to clinical application. Recently, Hu et al. demonstrated through molecular docking, interaction analyses, molecular dynamics simulations, and free energy calculations that complexes involving residues such as TRP14, PRO94, LYS120, ASN151, THR264, ASP260, and GLN298 exhibit strong stability and binding affinity [137]. They further identified 10 promising small molecules via DeLA-Drug analysis, providing theoretical support for the development of novel GPD1 inhibitors [137].

### Treatment plans targeting porphyrin metabolism

Enhanced lipogenesis in DCCs promotes porphyrin metabolism and the accumulation of PpIX [58]. This metabolic feature provides a rationale for 5-aminolevulinic acid photodynamic therapy (ALA-PDT) [58]. 5-ALA, synthesized from succinyl-CoA and glycine, serves as a porphyrin precursor that is converted into PpIX in mitochondria. PpIX can subsequently chelate Fe²⁺ to form heme [138] (Fig. 3). In addition to its role as a heme precursor, PpIX functions as a photosensitizer with a maximum excitation wavelength near 405 nm and peak emission around 635 nm [139]. Because DCCs accumulate high levels of PpIX, exposure to light of appropriate wavelength induces mitochondrial ROS generation, ultimately leading to cell death [58]. Clinically, 5-ALA is already employed in photodynamic diagnosis (ALA-PDD) [140] and ALA-PDT [141] for malignant tumors. Mitomycin C has been shown to induce dormancy in bladder cancer cells by arresting them in the G2/M phase, thereby enhancing PpIX accumulation and potentiating the cytotoxic effects of ALA-PDT [142]. Consequently, the combination of ALA-PDT and mitomycin C has been successfully applied in bladder cancer treatment [142]. However, the limited tissue penetration of visible light and the suboptimal pharmacokinetics of 5-ALA restrict its use in thicker lesions [143]. The development of novel 5-ALA derivatives with improved tumor selectivity and deeper tissue penetration holds promise for expanding the therapeutic utility of ALA-PDT.

## Conclusion and prospect

Investigating the metabolic characteristics of DCCs first requires their precise identification. Currently, commonly used markers for characterizing DCCs include p38 MAPK ^high^ /ERK ^low^, p21, and p27, among others [7, 8]. The next step involves dissecting metabolic reprogramming at the molecular level to construct a comprehensive map of metabolism in DCCs. Advanced techniques such as molecular imaging, single-cell transcriptome profiling, stable isotope tracing, and metabolomics offer powerful tools to achieve this. For example, integration of genome-scale metabolic models with transcriptomic data revealed that DCCs in acute lymphoblastic leukemia and colorectal cancer exhibit significant dysregulation of glucose, amino acid, and folate metabolism [144]. Another study, using stable isotope tracing combined with integrated transcriptomic, proteomic, and metabolomic analyses, unraveled the complex metabolic networks in dormant EOC cells and identified potential interventional targets [28].

For DCCs that rely predominantly on OXPHOS to maintain their dormant state, OXPHOS inhibitors undoubtedly represent an ideal class of therapeutic agents. However, the therapeutic efficacy of different classes of OXPHOS inhibitors varies widely across distinct DCC subtypes, and certain OXPHOS inhibitors only exert their pharmacological effects under specific conditions. IACS-010759, characterized by its broad antitumor spectrum and low propensity to induce drug resistance, is the most promising OXPHOS inhibitor for the targeted treatment of DCCs [116]. For DCCs with enhanced FAO, CD36 is an optimal metabolic target given its pleiotropic roles in promoting FAs uptake, lipid metabolic reprogramming, and immunosuppression [122]. However, similar to OXPHOS inhibitors, different CD36 inhibitors exhibit distinct subtype selectivity in their therapeutic effects. As the metabolic patterns of DCCs partially overlap with those of normal cells, insufficient target selectivity of these therapies readily leads to systemic toxicities, which is the root cause of failure for many relevant clinical trials. At present, our understanding of the regulatory mechanisms underlying metabolic reprogramming in DCCs remains no more than a fragmented puzzle. Completing this puzzle to identify DCC-specific metabolic regulatory targets holds great potential to improve the selectivity and safety of targeted therapies against DCCs.

Within the field of DCC metabolism, breast cancer is the most extensively studied cancer type to date. However, breast cancer is classified into four subtypes—Luminal A, Luminal B, Her2⁺ breast cancer, and TNBC [145], with substantial metabolic heterogeneity across cancer cells of different subtypes. Luminal breast cancer cells rely on OXPHOS for proliferation [49]. In their dormant state, these cells exhibit reduced OXPHOS and enhanced de novo lipogenesis, which protects them from ferroptosis; upon reactivation, OXPHOS and FAO are significantly upregulated [31, 61]. By contrast, Her2⁺ breast cancer and TNBC cells depend on glycolysis for proliferation [49]. When entering dormancy, these cells display attenuated glycolysis and upregulated OXPHOS and FAO, with glycolytic activity restored upon reactivation [43, 86]. At present, there is insufficient biological evidence to support metabolic-targeted therapies for dormant luminal breast cancer cells. Studies have reported that FASN inhibitors significantly reduce the viability of DCCs in vitro [62], but their in vivo antitumor efficacy remains to be further validated. Given the rapid disease progression and poor clinical prognosis of TNBC, several clinical trials have evaluated OXPHOS inhibitors or CD36 inhibitors for the treatment of this malignancy [106, 126]. However, the prevailing challenge remains dose-limiting toxicities arising from insufficient target selectivity. Optimizing drug delivery strategies to enhance the selectivity of therapeutic agents for the metabolic pathways of DCCs will substantially improve the safety profile and antitumor activity of these treatments.

Nanoparticles (NPs) have emerged as effective carriers that enhance drug delivery, bioavailability, and selectivity while reducing systemic toxicity [146]. Recent studies have demonstrated their potential for targeting DCCs. For example, Fernandes et al. developed iron oxide nanocubes (IONCs) coated with thermoresponsive polymers and loaded with doxorubicin; under magnetic hyperthermia (MHT), localized heating triggered drug release, effectively suppressing dormant CRC cells in vitro and in vivo [147]. Tsakiris et al. used lipid nanocapsules (LNCs) to co-deliver SN38 (targeting proliferative cells) and salinomycin (targeting DCCs) in CRC, achieving superior efficacy and reduced toxicity compared with free drugs [148]. Collectively, NPs hold great promise as delivery vehicles for metabolism-targeted therapeutics, capable of enhancing the selectivity of these agents for DCCs while reducing their systemic off-target toxicity.

In conclusion, research into tumor dormancy metabolism is still in its early stages. Current findings allow a preliminary outline of metabolic traits in DCCs, but the molecular mechanisms of their reprogramming remain incompletely understood—particularly in amino acid and nucleotide metabolism. The metabolism of DCCs is highly heterogeneous, and existing metabolic profiles cannot be universally applied across tumor types, as unique adaptations may yet be uncovered. Furthermore, the complexity of metabolic networks far exceeds current knowledge, with many interactions and their biological implications still undefined. With the integration of multi-omics technologies, future studies are expected to systematically unravel metabolic reprogramming of DCCs and construct comprehensive metabolic maps. These insights will accelerate the identification of therapeutic vulnerabilities, improve drug selectivity, and ultimately advance precision oncology.

## Data Availability

No datasets were generated or analysed during the current study.
